# The end of hunger: fertilizers, microbes and plant productivity

**DOI:** 10.1111/1751-7915.13973

**Published:** 2021-11-12

**Authors:** Hang‐Wei Hu, Qing‐Lin Chen, Ji‐Zheng He

**Affiliations:** ^1^ School of Agriculture and Food Faculty of Veterinary and Agricultural Sciences The University of Melbourne Parkville Vic. 3010 Australia; ^2^ ARC Hub for Smart Fertilisers The University of Melbourne Parkville Vic. 3010 Australia

## Abstract

It is a grand challenge to ensure the food security for a predicted world population of exceeding 9.7 billion by 2050, especially in an era of global climate change, land degradation and biodiversity loss. Current agricultural productions are mainly relying on synthetic chemical fertilisers to boost plant productivity but have undesirable effects on the environment and soil biodiversity. A promising direction in sustainable agriculture is to harness naturally occurring processes of beneficial plant‐associated microbiomes to ensure sustained crop production and global food security. Despite the significant progress made in the development of beneficial microbes as inoculants to enhance plant performance, challenges remain with the translation of knowledge of plant and soil microbiomes to successful microbial products in the agricultural sector. Here, we highlight how fertilizer technology should be renovated by harnessing microbiome‐based innovations to promote plant productivity and contribute to the end of hunger.

## Graphical abstract

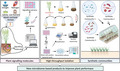

Given a projected world human population of near 10 billion by 2050 and higher frequencies of droughts and pathogen outbreaks attributable to climate change, an important question we have to address is how to increase food supply to feed the people (Tilman *et al*., 2011). In modern agriculture, synthetic fertilizers and agrochemicals (e.g., pesticides), together with high‐yielding cereal cultivars, significantly increased the global crop yield (Tillman *et al*., 2002) and reduced the poverty and hunger for a growing global population (Erisman *et al*., 2008). Undesirable detrimental environmental consequences of conventional agrochemicals, however, were recognised including increased environmental pollution, land degradation, chemical runoff and biodiversity losses. A great proportion of fertilizer nitrogen (exceeding 50% in some cases) is finally lost to the environment through ammonia volatilization and/or biological pathways such as nitrification and denitrification (Hu *et al*., 2017a,2017b), resulting in environmental pollution and a low crop nitrogen use efficiency. Enhanced efficiency fertilizers (e.g., controlled release fertilizers) and urease and nitrification inhibitors (Hu *et al*., 2017a,2017b; Shi *et al*., 2017) can mitigate such losses and improve the efficiency of fertilizers, but their performance is inconsistent across soil types and climates and some non‐biodegradable polymers in fertilizer coatings have adverse effects on the environment. Under the growing population pressure, an innovative and eco‐friendly resolution is crucially needed to achieve sustainable agricultural production.

In recent years, plant‐associated microbiomes (i.e., bacteria, archaea, fungi, viruses and protists colonizing plant hosts internally and externally) have emerged as a crucial component of sustainable agriculture for promoting soil health and ensuring agricultural sustainability (Batista and Singh, 2021; Chen *et al*., 2021a; Mitter *et al*., 2021; Sun *et al*., 2021). These plant‐associated microbes can perform a wide range of life‐beneficial functions, including plant nutrient acquisition, immune development and plant tolerance of multiple abiotic and biotic stresses (Hoeksema *et al*., 2018; Singh *et al*., 2020). There is growing interest, therefore, in developing novel microbiome‐based technology to harness the beneficial plant−microbe interactions to sustainably promote crop performance under constrained conditions such as drought or pathogen infestation. One of a few successful cases that capitalize on microbiome‐based technologies is biofertilizers, which are expected to be a highly sustainable substitute for conventional chemicals and a pivotal component in integrated agroecosystem management. Biofertilizers are defined as bio‐based substances that contain microbes or other living organisms that can provide optimum nutrients to crop, protect soil biodiversity, and contribute to plant growth and crop yield. It is estimated that the global market value of agricultural biologicals, including biofertilizers, biocontrol agents, and biostimulants, will exceed US$18.9 billion by 2025, driven by concerns toward environmental safety, and increasing regulations on chemical usage (e.g., some key pesticides) and maximum residue levels.

A wide range of agriculturally beneficial microbes (e.g., plant growth promoting rhizobacteria, N_2_‐fixing rhizobacteria, phosphorus and potassium solubilising microorganisms, mycorrhizal fungi, disease suppressive microbial community, and stress tolerance microbes) can be exploited as inoculants for biofertilizers to enhance plant nutrition. These beneficial microbes have positive effects on plant growth through enhancing nutrient availability, biosynthesis of phytohormones, stimulation of root system architecture, and protecting host plants from biotic or abiotic stresses (Niu *et al*., 2018; Trivedi *et al*., 2020). Plant non‐pathogenic endophytes, microorganisms that colonize internal plant tissues (e.g., roots, leaves, flowers, stems, fruits and seeds), are also important to the health and performance of their host and have received growing attention in recent years (Compant *et al*., 2021; Martinez‐Romero *et al*., 2021). Expression of various nitrogen‐cycling genes was reported in rice roots, indicating the role of endophytes in the processes of nitrogen fixation, nitrification and denitrification (Sessitsch *et al*., 2012). Other beneficial functions of plant endophytes include phosphate mobilization, suppression of pathogens, plant defense induction, secreting hormones to promote plant health and promoting plant resistance to environmental stresses (Compant *et al*., 2021). A comprehensive understanding of the interplay between plants and endophytes, therefore, would open up new opportunities to apply endophytic communities for sustainable crop production.

Exploitation of plant beneficial microbes as inoculants for cereals, such as maize, wheat and rice, is an emerging strategy to boost the sustainable agriculture, but their functionality and persistence in field is generally inconsistent owing to underdeveloped inoculation technology, out‐competition by indigenous microbes, suboptimal rhizosphere colonisation and lack of stringent host‐specificity (Haskett *et al*., 2021). To date, most of previous studies have mainly focused on the roles of specific groups of plant‐associated microorganisms on plant health (Xu *et al*., 2021), which are often constrained by the low diversity of the microbial inoculants that can establish in the complex microbiome (Li *et al*., 2021). To overcome these shortcomings, recent studies have started to use synthetic communities (SynComs) as a small‐scale selection of plant associated microbes to promote soil health and contribute to plant growth and yield (Chen *et al*., 2021b; Trivedi *et al*., 2021). The SynComs approach has advantage of improving the diversity of inoculated strains with a better predicted performance than single strain inoculation under the field conditions (Compant *et al*., 2021). However, we still lack a good understanding of how plants recruit the army of beneficial microbes under various abiotic and biotic stresses, and how these microbes communicate with each other and with plant hosts to support crop performance and host fitness (Wei *et al*., 2019). A holistic microbiome perspective to decipher the biochemical and genetic mechanisms that govern the establishment of plant‐beneficial microbes is a prerequisite to increase potential biotic repertoire to be applicable in sustainable plant management.

The plant−microbe interaction is a two‐way communication process, mediated by a large number of diverse chemical and molecular signalling molecules such as sugars, organic acids, proteins, peptides, lipids, phytohormones and secondary metabolites in plant rhizosphere soils (Venturi and Keel, 2016; Trivedi *et al*., 2020; Macabuhay *et al*., 2021). Multi‐omics approaches enable characterization of the taxonomy and beneficial properties of core microbial communities colonising the different plant compartments (Macabuhay *et al*., 2021; Sun *et al*., 2021; Trivedi *et al*., 2021), but a combination of mass spectroscopy and bioanalytical chemistry tools is urgently needed to unravel the signalling pathways that drive microbial interactions toward better plant performance and resilience to stresses (Bauermeister *et al*., 2021). A recent study attributed the variances in nitrogen use efficiency among rice varieties to the recruitment of nitrogen‐cycling microorganisms in rhizosphere soils, with *Oryza indica* attracting more efficient nitrogen‐cycling microbes in the rhizosphere than *Oryza japonica* varieties (Zhang *et al*., 2019). Plants secrete specialized secondary metabolites, such as coumarins for *Arabidopsis thaliana*, which can enhance iron mobilization and generate reactive oxygen species to inhibit root bacterial communities that compete with plants for iron (Voges *et al*., 2019). Tomato plant with high resistance to *Ralstonia solanacearum* (a pathogenic bacterium causing tomato wilt) enriches *Flavobacterium* spp. in the rhizosphere to suppress pathogens (Kwak *et al*., 2018).

Under nitrogen‐limited conditions, plants can sense and respond to the distribution and availability of external nutrient pools (Gent and Forde, 2017), through modulating the transport of amino acids, plant hormones, and auxins to regulate root and selectively recruit a beneficial microbiome (Trivedi *et al*., 2020). Under phosphorus‐ or iron‐limited conditions, the biosynthesis and root exudation of scopoletin is induced by the transcription factor MYB72 and the β‐glucosidase BGLU42, which are recruited to DNA regulatory sequences resulting in significant metabolic changes that promote the proliferation of beneficial microbes that alleviate stress (Trivedi *et al*., 2020). These metabolites triggered by environmental stresses act as signalling molecules to tailor the plant rhizosphere microbiome and improve plant performance (Voges *et al*., 2019). It is imperative, therefore, to identify the core group of crop‐specific plant signalling molecules under various environmental stresses, followed by the extraction and selection of the signal molecules with the capacity to attract, promote or maintain activities of beneficial microbes in the rhizosphere (Fig. 1). Upon incorporation of these signalling molecules in biofertilizers, the new products would release these microbial chemicals to enhance beneficial plant–microbe interactions, with positive consequences for plant nutrient acquisition and resistance to drought, pathogens and diseases. Recent studies have utilized synthetic host‐specific plant‐to‐bacteria signalling to establish exclusive coupling of plant−microbe interactions in the field (Geddes *et al*., 2019).

**Fig. 1:**
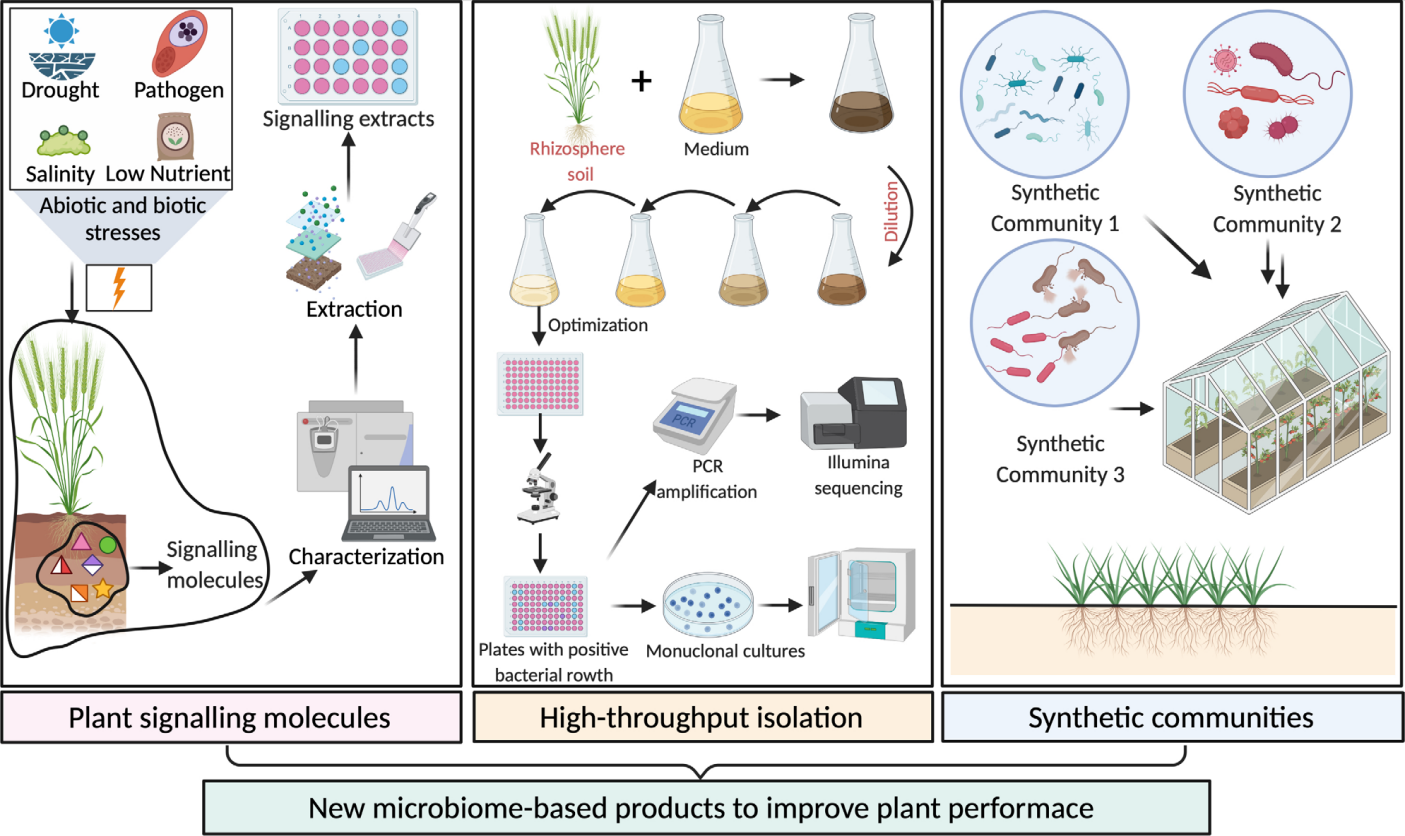
Conceptual diagrams to harness the plant−microbe interactions for the development of new microbial products to improve plant performance. We propose to identify the core group of crop‐specific plant signalling molecules under various environmental stresses and to extract the selected signalling molecules with the capacity to promote activities of beneficial microbes in the rhizosphere. The high‐throughput isolation approach can be used to isolate plant beneficial microbes in a high‐throughput manner, and the isolated bacterial cultures can be used to create various synthetic communities to test their effects on plant performance in controlled and field experiments.

In order to improve the diversity of microbial inoculants for a better field performance, a promising direction to innovate the design of new biofertilizers is to generate a tailor‐made core microbiome transfer therapy for different crops under specific environmental and agricultural settings (Gopal *et al*., 2013). We propose to use the high‐throughput isolation of plant beneficial microbes for new biofertilizer development (Zhang *et al*., 2021). This high‐throughput isolation approach could allow establishing a taxonomically comprehensive bacterial culture collection from the plant rhizosphere soil (Wippel *et al*., 2021), which is essential for the functional and mechanistic studies of bacteria with strain‐specific resolution. The high‐throughput isolation method uses a limited dilution of the rhizosphere sample with selective media in 96‐well cell culture plates, followed by a barcode PCR system to characterize pure bacterial strains (Fig. 1). The 16S rRNA gene sequences in bacterial cultures can be cross‐referenced with 16S rRNA gene sequences generated from plant rhizosphere samples under controlled conditions, allowing for targeted isolation of bacteria of interest (e.g., key bacterial taxa that best positively predict plant performance). The isolated bacterial cultures can be used to design SynComs with different complexity to identify the most efficiency strain or combination of strains (Fig. 1). The effects of synthetic community on the plant growth promotion will be evaluated based on the observations, and the contribution of each strain and the most efficiency synthetic combination can be identified. Application of these SynComs with varying complexity (i.e., different numbers of cultured strains) to axenic plants can unravel the physiological characteristics of the bacterial community, especially under defined abiotic or biotic stresses.

The increasing emphasis on sustainable agriculture has opened up a wealth of opportunities to integrate microbiome‐based products into modern agricultural management programmes for long‐term sustained benefits. We envision that embracing a holistic view of plant−microbiome interactions for developing new microbial products that are tailored and integrative in diverse agricultural systems will result in a sustainable agriculture of important crops while increasing food production at the same time. These multidisciplinary innovations in microbial products will lead to better integrated efforts to develop appropriate strategies to address the challenges of achieving food security in more efficient and more profitable ways.

## Conflict of interest

None declared.
